# Rapid expansion of TILs from patients with glioma and recognition of autologous tumor

**DOI:** 10.1186/2051-1426-2-S3-P27

**Published:** 2014-11-06

**Authors:** Liu Zhenjiang, Qingda Meng, Oscar Persson, Bartek Jiri, Thomas Poiret, Lalit Rane, Elena B Rangelova, Ernest Dodoo, Markus Maeurer

**Affiliations:** 1 Karolinska Insitutet, Stockholm, Sweden; 2 Karolinska Insitutet, Karolinska University Hospital, Stockholm, Sweden; 3 Dept. of Neurosurgery, Karolinska University Hospital, Stockholm, Sweden

## Background

Tumor-infiltrating T cells (TIL) may represent a viable source of T cells for the biological treatment of patients with tumors of the central nervous system. We established a rapid TIL expansion protocol for patients with glioblastoma, and tested the recognition of short-term expanded tumor autologous tumor cell lines defined by cytokine production from responding T cells.

## Material and methods

Glioma tumor tissue was obtained from 15 patients with glioblastoma, tumor cell lines were established and TIL could be successfully expanded in 15/15 cases using a cytokine cocktail IL-2/IL-15/IL-21, OKT-3 and irradiated, allogeneic feeder cells. Intracellular Cytokine Staining (ICS) was used to detect antigen-specific immune responses. Autologous tumor cells or TAAs (NY-ESO-1, Survivin and EGFRvIII peptides) were co-cultured with TILs for 6 hours in the presence of Brefeldin A as well as in medium (negative control), or PMA+Ionomycin (positive control). CD3, CD4 and CD8 markers were combined with either IL-2, IL17, TNFalpha, IFNgamma production or 41-BB expression. VB family composition, exhaustion/activation as well as differentiation markers were tested by flow cytometry.

## Results

15/15 TIL could be successfully expanded (up to 10e10 cells) using IL-2, IL-15 and IL-21. The majority of TIL exhibited a mixture of CD4+, CD8+, as well as CD3+ (TCRalpha/beta) CD4-CD8- T cells with an CD45RA-CCR7+ phenotype. TILs showed low frequencies of exhaustion markers, i.e. PD-1 (in CD8+ TILs: median: 3.03%, mean: 3.40%, in CD4+ TILs: median: 2.10%, mean: 7.73%), TIM-3 (in CD8+ TILs: median: 0.30%, mean: 1.21%, in CD4+ TILs: median: 0.70%, mean: 1.00%), CTLA-4 (in CD8+ TILs: median: 1.47%, mean: 1.73%, in CD4+ TILs: median: 0.04%, mean: 0.07%) and LAG-3 (in CD8+ TILs: median: 8.87%, mean: 30.00%, in CD4+ TILs: median: 1.21%, mean: 1.25%). Some TIL cultures exhibited preferential usage of VB families (i.e up to 60% VB22 or VB1 in CD4+ or CD8+ TIL). TIL from individual patients exhibited NY-ESO-1 specificity up to 2% in CD4 and CD8+ T cells, yet up to 25% IFN and TNF production directed against autologous tumor cells, defined by ICS. Whole genome sequencing is currently performed in gliomas to subsequently test for subsequent TIL recognition.

## Conclusion

TIL from glioma samples can be reliably and successfully expanded in IL-2/IL-15 and IL-21, they exhibit a Th1-cytokine production pattern and recognize autologous tumor cells ex vivo. Glioma - reactive TIL represent a viable source the cellular therapy for patients with glioblastoma and a Phase I safety trial is currently prepared at Karolinska.

## Consent

Written informed consent was obtained from the patient for publication of this abstract and any accompanying images. A copy of the written consent is available for review by the Editor of this journal.

